# Supplementary material for: Establishing functional giant Dictyostelium cells reveals front–rear polarity in intracellular signaling
Source: Commun Biol. 2026 Jan 21;9:71. doi: 10.1038/s42003-025-09505-7 (PMC12824319; doi:10.1038/s42003-025-09505-7)
Supplement: Supplementary file 2 — Supplementary Materials [file 42003_2025_9505_MOESM2_ESM.pdf]

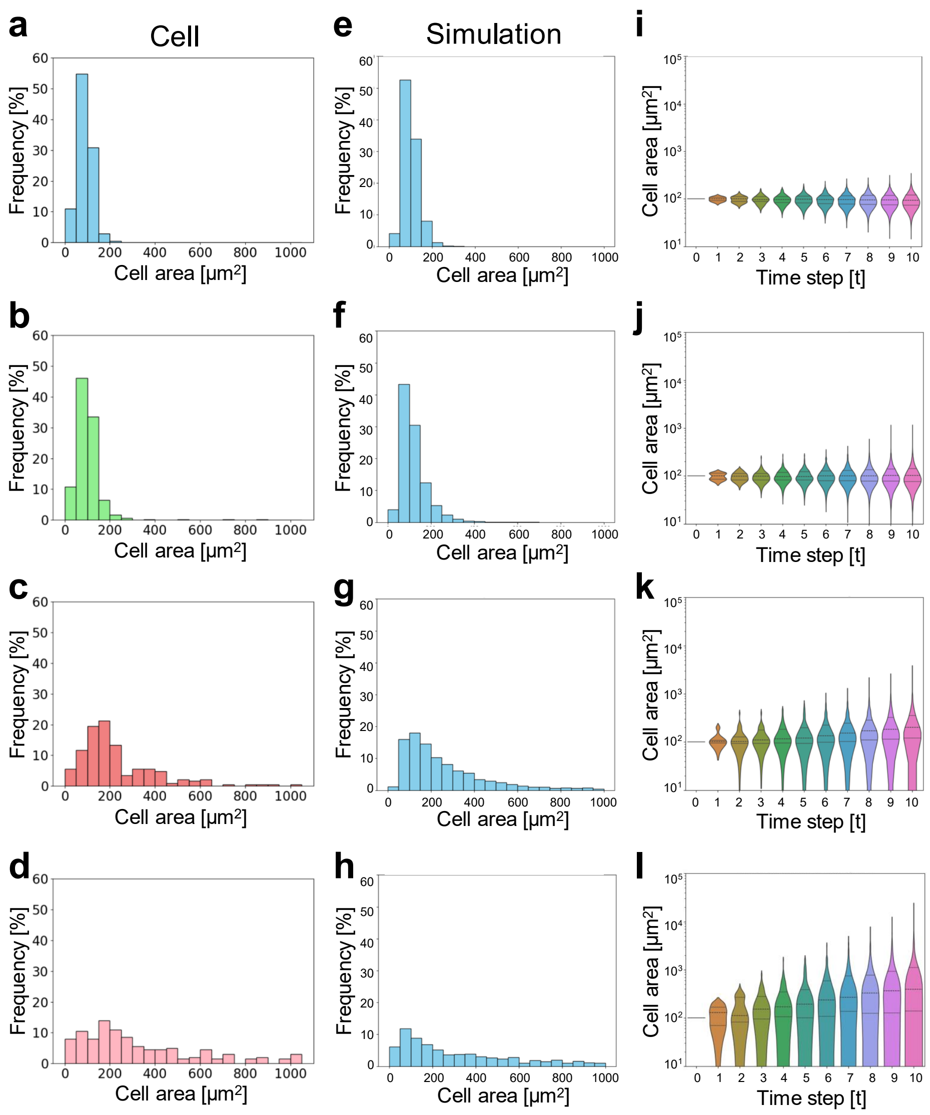

Supplementary Figure 1: Distribution and temporal changes of cell size in simulations and experiments.

a–d, Histograms showing the distribution of cell sizes from the experiments. a–d show the same data as presented in Fig. 1a–d, respectively. **e–h**, Simulation results of cell size distributions based on a discrete-time stochastic model are shown. These are similar to the experimental data presented in Fig. 1a–d. All simulations represent the state after 10 time steps. In each simulation, the initial number of cells N_0 was set to 10, and the initial size S_0 was 100. The minimum size required for division (S_{min}) was fixed at 50. Panel e shows the case where the probability of cell division P_{div} was 1.00 and the division ratio p ranged from 0.40–0.60. Panel f represents the condition with P_{div} = 0.95 and the same division ratio range (0.40–0.60). Panel g depicts the case with a lower division probability of 0.80, maintaining the same p range. Panel h further decreases P_{div} to 0.60 and applies a broader division ratio range from 0.20–0.80. **i–l**, The progression of cell size distributions over each time step in the simulations shown in panels e–h, respectively.

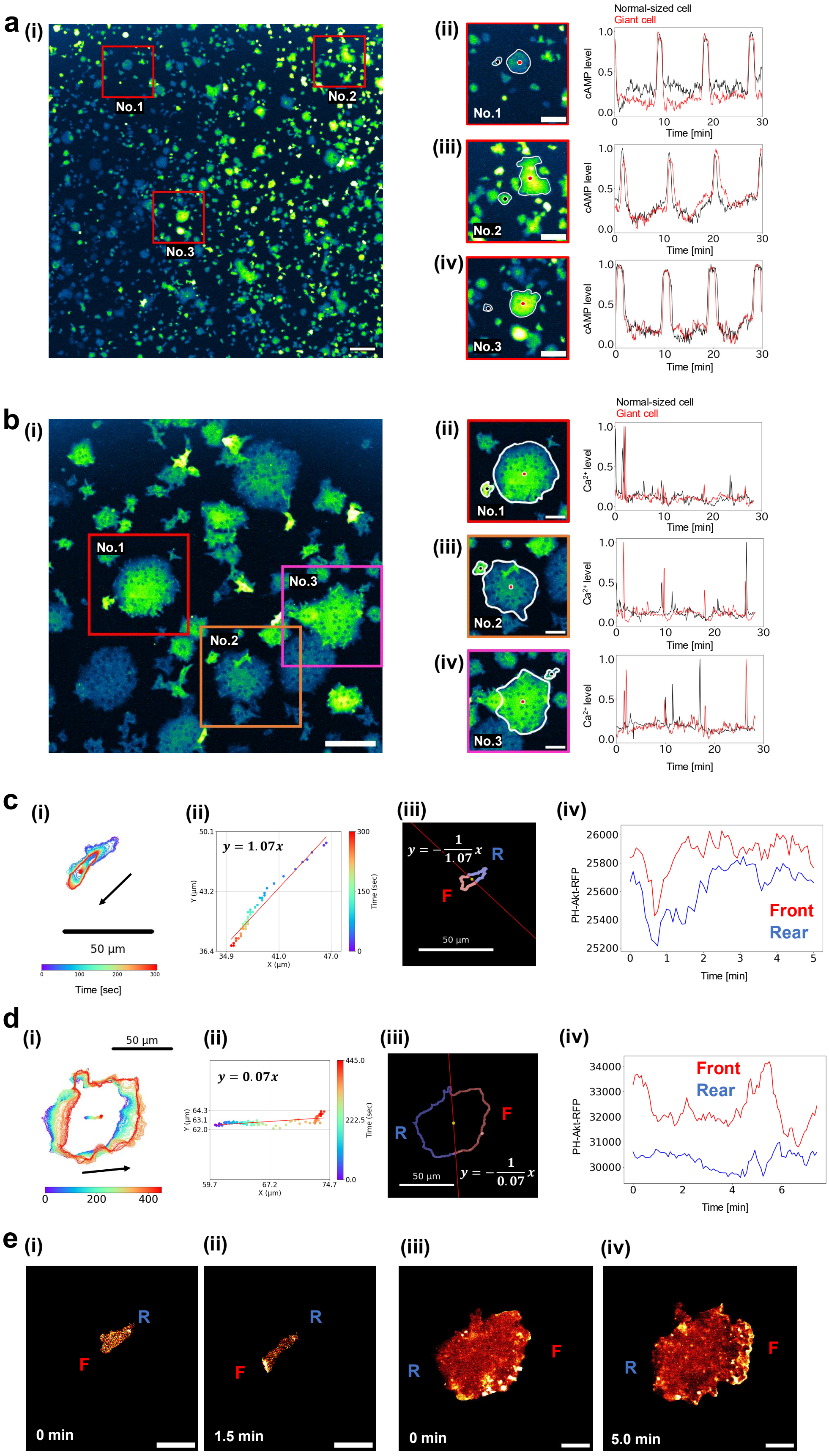

Supplementary Figure 2: Comparison of cell dynamics in the cAMP relay between normal-sized and giant cells.

a, AX2 cells expressing Flamindo2 were cultured in HL5 medium with 10 μM blebbistatin

under shaking conditions for 8 days. After the cells were washed and starved in DB, fluorescence imaging was performed using confocal microscopy (10× objective, 5 sec intervals). (i) A fluorescence image showing the entire field of view. Scale bar: 100 μm. Regions where giant and normal-sized cells were adjacent are highlighted in red boxes. (ii–iv) cAMP signal dynamics in individual cells (ROIs No.1, 2, and 3). Left panels: Enlarged views of the red boxes shown in (i). Scale bar: 50 μm. Black dots: ROIs in normal-sized cells (diameter: 5.5 μm). Red dots: ROIs in giant cells (diameter: 5.5 μm). Right panels: Plots of normalized cAMP levels over time. Horizontal axis: time [min]; vertical axis: normalized cAMP level. Black lines: normal-sized cells; red lines: giant cells. **b**, AX2 cells expressing GCaMP6s were cultured in HL5 medium with 10 μM blebbistatin under shaking conditions for 5 days. After the cells were washed and starved in DB, fluorescence imaging was performed using confocal microscopy (40× objective, 5 sec intervals). (i) A fluorescence image showing the entire field of view. Scale bar: 50 μm. Regions where giant and normal-sized cells were adjacent are highlighted in red, orange, and magenta boxes. (ii–iv) Ca^{2+} signal dynamics in individual cells (ROIs No.1, 2, and 3). Left panels: Enlarged views of the red boxes shown in (i). Scale bar: 20 μm. Black dots: ROIs in normal-sized cells (diameter: 5.5 μm). Red dots: ROIs in giant cells (diameter: 5.5 μm). Right panels: Plots of normalized Ca^{2+} levels over time. Horizontal axis: time [min]; vertical axis: normalized Ca^{2+} level. Black lines: normal-sized cells; red lines: giant cells. **c, d**, AX2 cells expressing PH(Akt)-RFP were cultured in HL5 medium under static conditions (c) or with 10 μM blebbistatin under shaking conditions for 6 days (d). After the cells were washed and starved in DB, fluorescence imaging was performed using confocal microscopy (40× objective, 5 sec intervals). (i) Centroid trajectories of normal-sized (c) and giant (d) cells. The contours of the cells and their centroids are overlaid. Arrows indicate the direction of migration. Scale bars: 50 μm. (ii) Linear approximation of the centroid trajectories. (iii) Definition of the analysis range for front–rear polarity. The front and rear of the cells are defined by a line perpendicular to the direction of movement that passes through the cell centroid. Red: cell front; Blue: cell rear. Contour width: 1.4 μm. Bars: 50 μm. (iv) Localization of PH(Akt)-RFP in the cells. Vertical axis: PH(Akt)-RFP localization; Horizontal axis: time [min]. Red: cell front; Blue: cell rear. **e,** (i), (ii) Fluorescence images showing the localization of PH(Akt)-RFP in the normal-sized cell shown in (c). (iii), (iv) Localization of PH(Akt) in the giant cell shown in (d). Each pair shows the initial field of view and the peak of PH(Akt) localization at the cell front. Elapsed time from imaging start is indicated at the lower left. Bars: 20 μm.

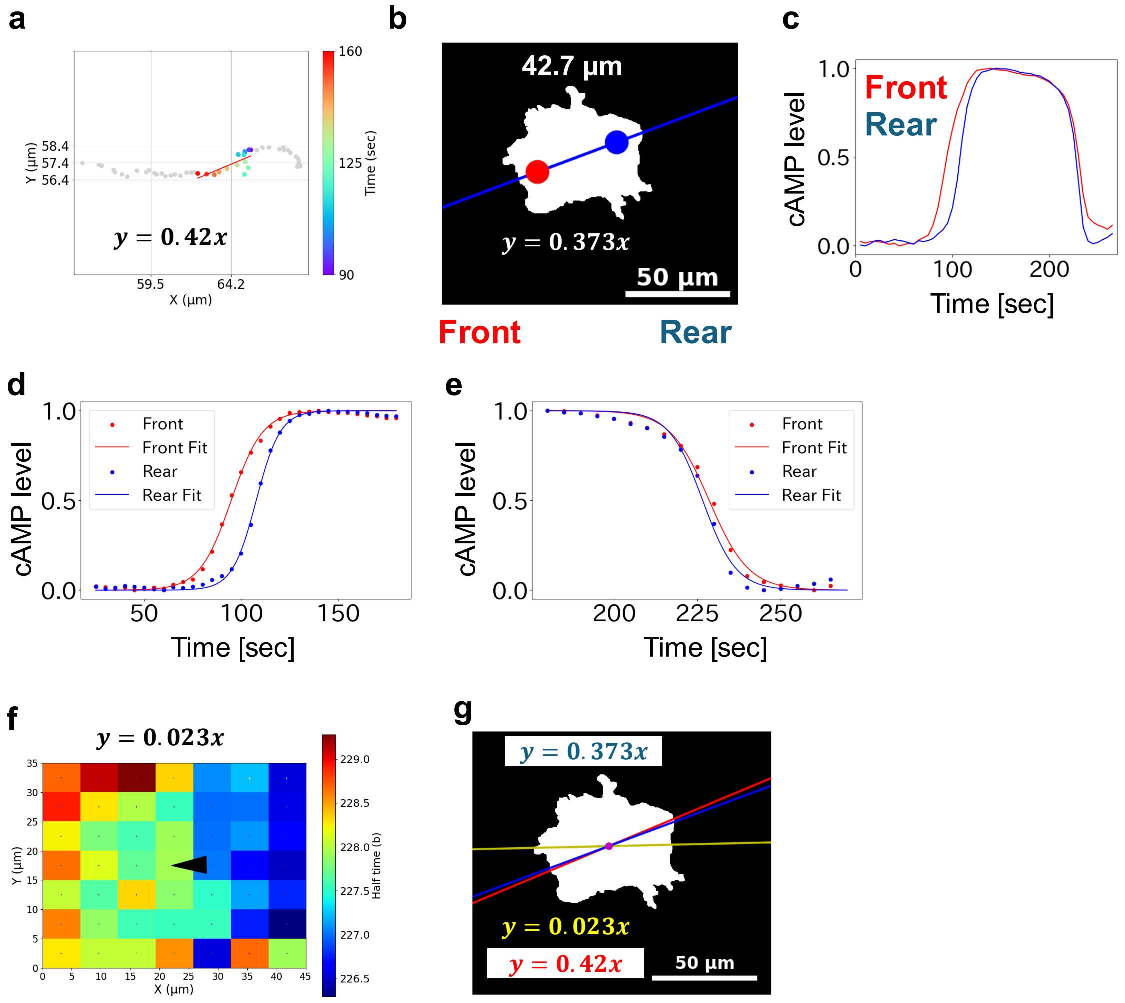

Supplementary Figure 3: Spatiotemporal analysis of cAMP increase and decrease. Analysis of the giant cell in Fig. 2. **a**, Linear approximation of the centroid trajectory. The centroid movement during 90–160 sec was approximated by a straight line. **b**, Definition of the front and rear regions for analysis. Using the slope (0.373) obtained from the cAMP increase gradient direction, the front and rear points were defined at ±20 µm in the x direction and ±0.373 × 20 µm in the y direction from the centroid. Red and blue circles indicate the front and rear measurement regions, respectively (diameter: 11 µm). **c**, Time-course of cAMP levels in the front (red) and rear (blue) regions. cAMP levels were calculated by inverse normalization. **d, e**, Sigmoid fittings of cAMP increase (d) and decrease (e) in the front (red) and rear (blue) regions. **f**, Heatmap of the half-time parameter of cAMP decrease for determining the intracellular cAMP gradient direction. Δx = 6.429 µm; Δy = 5.000 µm. Each arrow represents a local gradient vector, and the central arrow indicates the representative overall gradient slope. **g**, Comparison of cell migration direction with cAMP increase and decrease gradient directions. Red: cell migration direction; Blue: cAMP increase gradient; Yellow: cAMP decrease gradient.

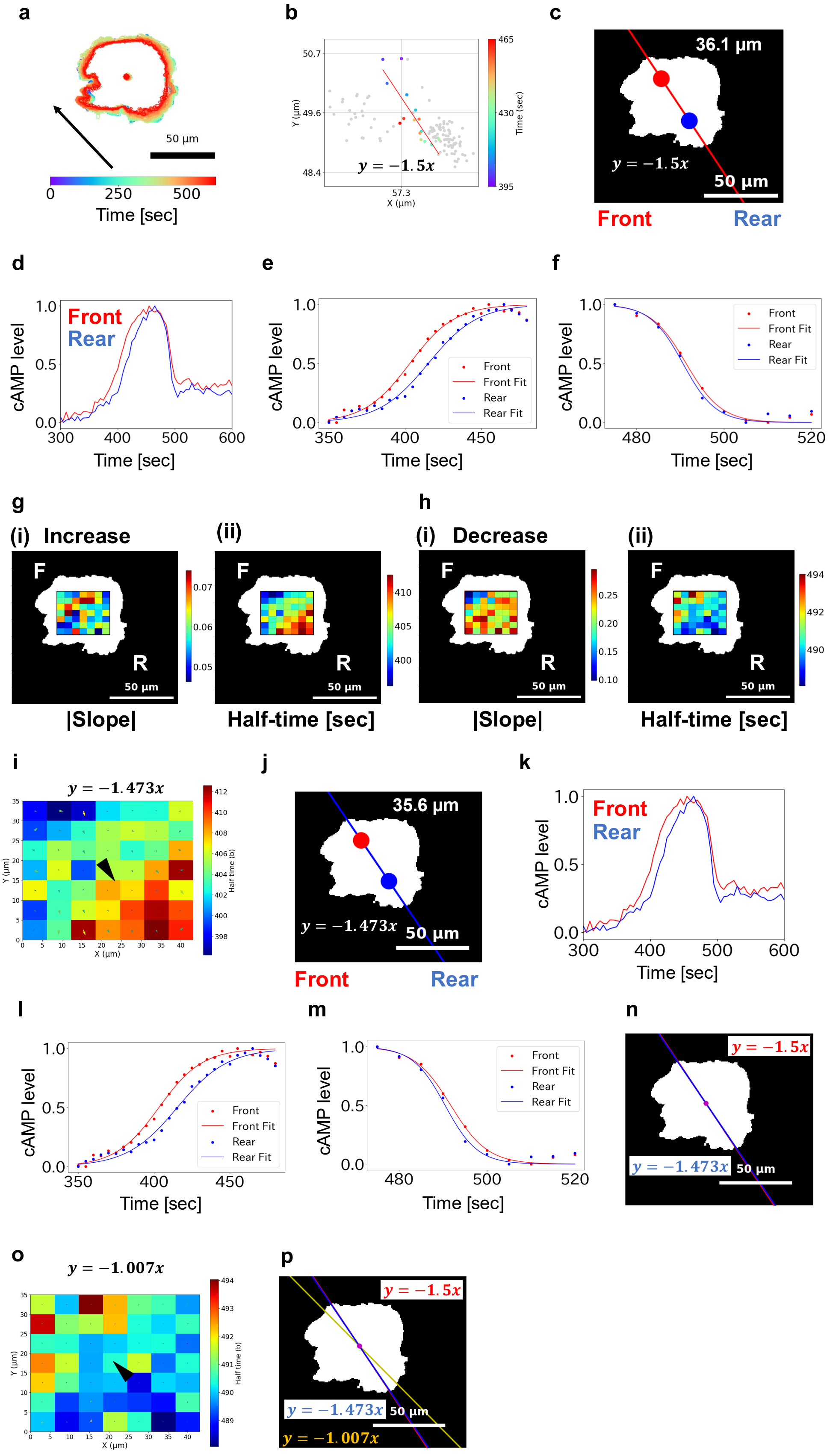

Supplementary Figure 4: Spatiotemporal analysis of cAMP in another cell.

AX2 cells expressing Flamindo2 were cultured with 10 µM blebbistatin under shaking conditions for 8 days and then collected at 1×10^6 cells/mL. After the cells were washed and

starved with DB, confocal imaging was then initiated (40× objective, 5 sec intervals). **a**, Cell outline overlaid with the centroid trajectory. An arrow indicates the direction of migration. **b**, Linear approximation of the centroid trajectory between 395 and 465 sec. **c**, Definition of front (red) and rear (blue) regions for analysis based on the fitted trajectory. The regions were positioned ±10 μm from the centroid along the fitted line with a slope of -1.5 (diameter: 11 μm). **d**, Temporal changes in normalized inverted cAMP intensity in the front (red) and rear (blue) regions (x-axis: time [s]; y-axis: normalized inverted cAMP intensity). **e**, Sigmoid fitting of cAMP increase (red, front; blue, rear). **f**, Sigmoid fitting of cAMP decrease. **g**, (i) Heatmap of the slope parameter during cAMP increase; (ii) heatmap of the half-time parameter during cAMP increase. **h**, (i) Heatmap of the slope parameter during cAMP decrease; (ii) heatmap of the half-time parameter during cAMP decrease. **i**, Heatmap of the half-time parameter of cAMP increase. Δx = 6.143 μm; Δy = 5.000 μm. **j**, Based on the gradient direction of cAMP increase, front (red) and rear (blue) regions were defined along the fitted line at ±10 μm from the centroid with a slope of -1.473 (diameter: 11 μm). **k**, Temporal changes in normalized inverted cAMP intensity in the front (red) and rear (blue) regions. **l**, Sigmoid fitting of cAMP increase (red, front; blue, rear). **m**, Sigmoid fitting of cAMP decrease. **n**, Comparison of the direction of cell migration (red) with the gradient direction of cAMP increase (blue). **o**, Gradient direction of cAMP decrease determined in the same manner. The slope is -1.007. **p**, Comparison of the directions of cell migration (red), cAMP increase gradient (blue), and cAMP decrease gradient (yellow).

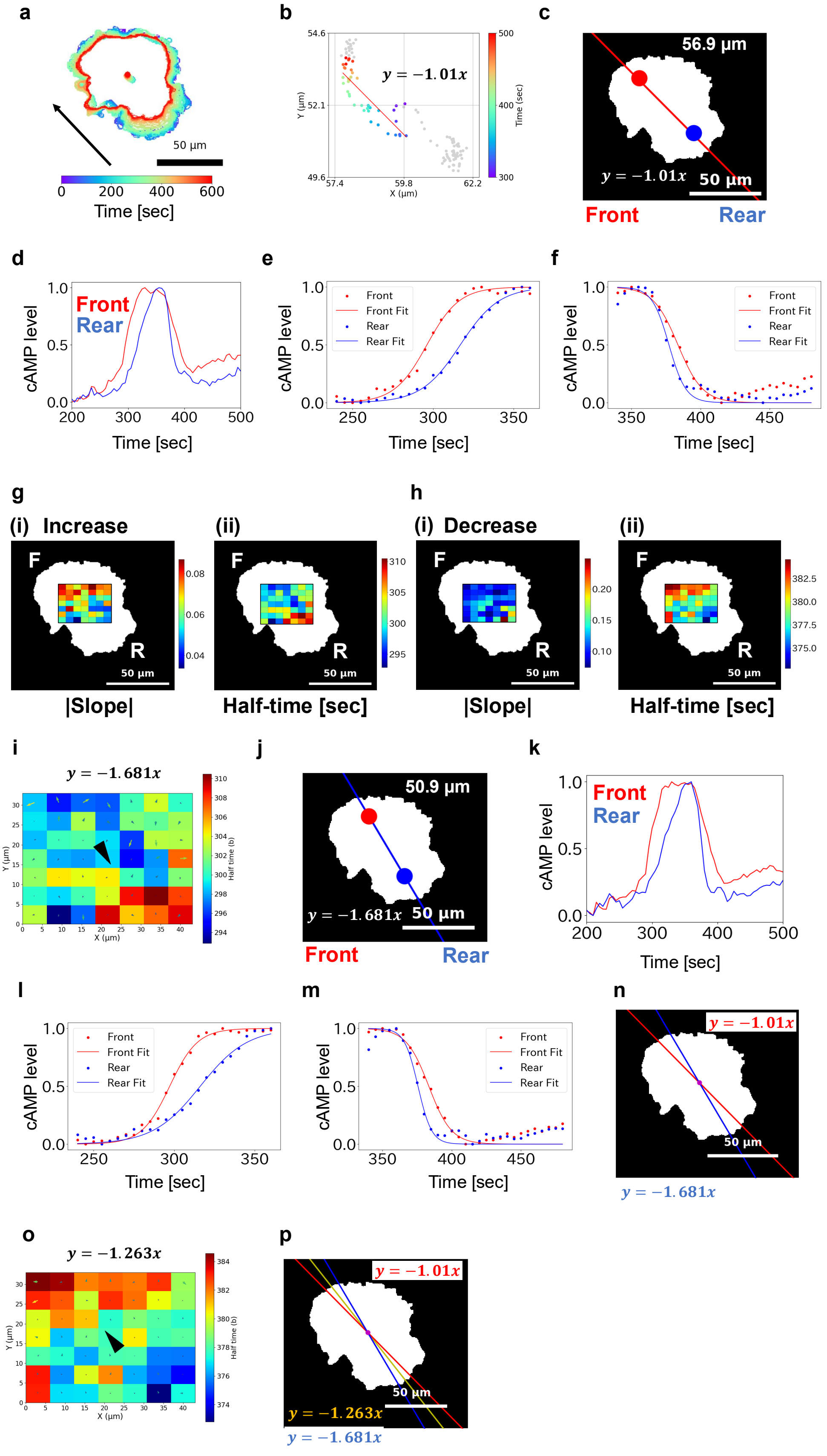

Supplementary Figure 5: Spatiotemporal analysis of cAMP in a different cell.

AX2 cells expressing Flamindo2 were cultured with 10 µM blebbistatin under shaking conditions for 8 days and then collected at 1×10^6 cells/mL. After the cells were washed and

starved with DB, confocal imaging was then initiated (40× objective, 5 sec intervals). **a**, Cell outline overlaid with the centroid trajectory. An arrow indicates the direction of migration. **b**, Linear approximation of the centroid trajectory between 300 and 500 sec. **c**, Definition of front (red) and rear (blue) regions for analysis based on the fitted trajectory. The regions were positioned ±20 μm from the centroid along the fitted line with a slope of −1.01 (diameter: 11 μm). **d**, Temporal changes in normalized inverted cAMP intensity in the front (red) and rear (blue) regions (x-axis: time [s]; y-axis: normalized inverted cAMP intensity). **e**, Sigmoid fitting of cAMP increase (red, front; blue, rear). **f**, Sigmoid fitting of cAMP decrease. **g**, (i) Heatmap of the slope parameter during cAMP increase; (ii) heatmap of the half-time parameter during cAMP increase. **h**, (i) Heatmap of the slope parameter during cAMP decrease; (ii) heatmap of the half-time parameter during cAMP decrease. **i**, Heatmap of the half-time parameter of cAMP increase. Δx = 6.143 μm; Δy = 4.714 μm. **j**, Based on the gradient direction of cAMP increase, front (red) and rear (blue) regions were defined along the fitted line at ±13 μm from the centroid with a slope of −1.681 (diameter: 11 μm). **k**, Temporal changes in normalized inverted cAMP intensity in the front (red) and rear (blue) regions. **l**, Sigmoid fitting of cAMP increase (red, front; blue, rear). **m**, Sigmoid fitting of cAMP decrease. **n**, Comparison of the direction of cell migration (red) with the gradient direction of cAMP increase (blue). **o**, Gradient direction of cAMP decrease determined in the same manner. The slope is -1.263. **p**, Comparison of the directions of cell migration (red), cAMP increase gradient (blue), and cAMP decrease gradient (yellow).

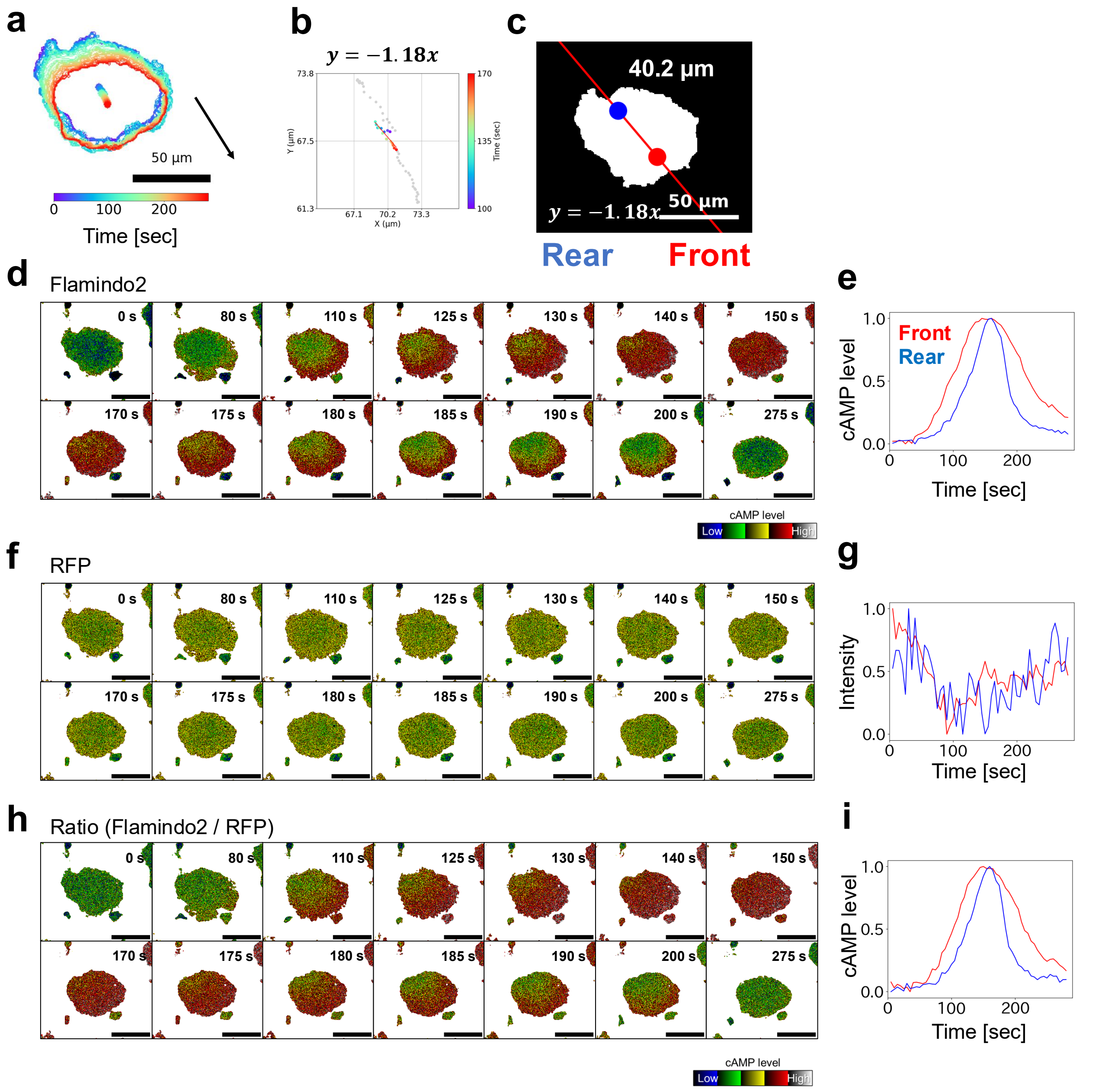

Supplementary Figure 6: Detailed analysis of intracellular cAMP dynamics with Flamindo2-RFP ratiometric imaging.

AX2 cells expressing Flamindo2-RFP were cultured with 10 µM blebbistatin under shaking conditions for 7 days. After the cells were washed and starved with DB, imaging was performed using confocal microscopy (40× objective, 5 sec intervals). **a**, Cell outline overlaid with the centroid trajectory. An arrow indicates the direction of migration. **b**, Linear approximation of the centroid trajectory between 100 and 170 sec. **c**, Definition of front (red) and rear (blue) regions for analysis based on the fitted trajectory. The regions were positioned ±13 µm from the centroid along the fitted line with a slope of −1.18 (diameter: 11 µm). **d**, Time-lapse fluorescence images (Flamindo2 channel) of the cell shown in (a). The number in the top right indicates the elapsed time from the start of imaging. Scale bar: 50

μm. **e**, Temporal changes in cAMP levels at the front (red) and rear (blue), based on Flamindo2 signal intensity. Data are shown as inverted and normalized fluorescence values. **f**, Time-lapse fluorescence images from the RFP channel of the same cell. **g**, Fluorescence intensity changes in the RFP channel at the front (red) and rear (blue), representing brightness variation caused by cell movement. **h**, RFP/Flamindo2 ratio images to reduce motion artifacts. Representative time-lapse images showing corrected intensity at each time point. **i**, Corrected cAMP level changes at the front (red) and rear (blue) based on the RFP/Flamindo2 ratio, presented as normalized values.

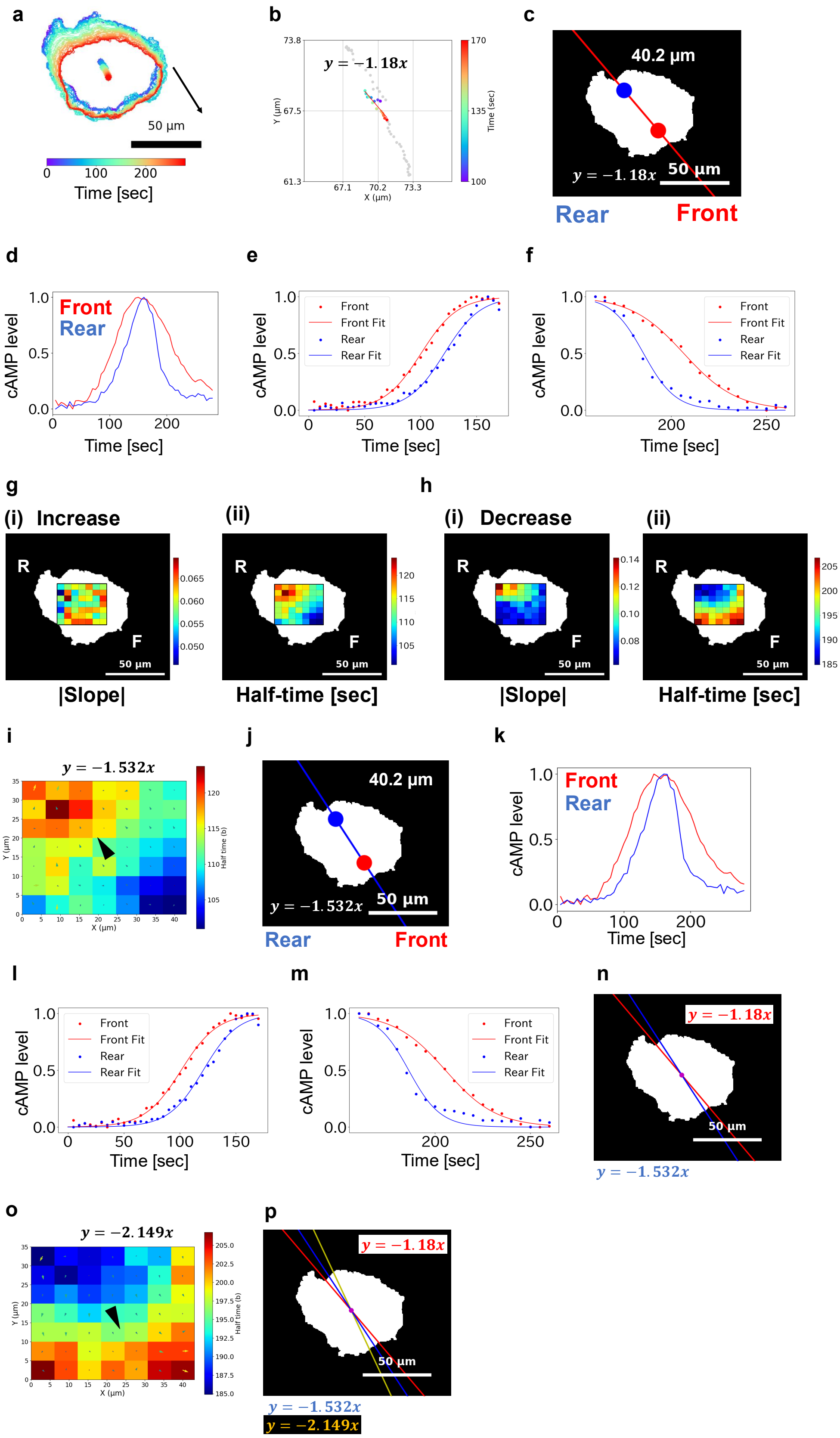

Supplementary Figure 7: Spatiotemporal analysis of cAMP with Flamindo2-RFP in a representative cell.

AX2 cells expressing Flamindo2-RFP were cultured with 10 µM blebbistatin under shaking

conditions for 7 days and then collected at 1×10^6 cells/mL. After the cells were washed and starved with DB, confocal imaging was then initiated (40× objective, 5 sec intervals). **a**, Cell outline overlaid with the centroid trajectory. An arrow indicates the direction of migration. **b**, Linear approximation of the centroid trajectory between 100 and 170 sec. **c**, Definition of front (red) and rear (blue) regions for analysis based on the fitted trajectory. The regions were positioned ±13 μm from the centroid along the fitted line with a slope of -1.18 (diameter: 11 μm). **d**, Temporal changes in normalized intensity ratio in the front (red) and rear (blue) regions (x-axis: time [s]; y-axis: normalized intensity ratio). **e**, Sigmoid fitting of cAMP increase (red, front; blue, rear). **f**, Sigmoid fitting of cAMP decrease. **g**, (i) Heatmap of the slope parameter during cAMP increase; (ii) heatmap of the half-time parameter during cAMP increase. **h**, (i) Heatmap of the slope parameter during cAMP decrease; (ii) heatmap of the half-time parameter during cAMP decrease. **i**, Heatmap of the half-time parameter of cAMP increase. Δx = 6.143 μm; Δy = 5.000 μm. **j**, Based on the gradient direction of cAMP increase, front (red) and rear (blue) regions were defined along the fitted line at ±11 μm from the centroid with a slope of -1.532 (diameter: 11 μm). **k**, Temporal changes in normalized intensity ratio in the front (red) and rear (blue) regions. **l**, Sigmoid fitting of cAMP increase (red, front; blue, rear). **m**, Sigmoid fitting of cAMP decrease. **n**, Comparison of the direction of cell migration (red) with the gradient direction of cAMP increase (blue). **o**, Gradient direction of cAMP decrease determined in the same manner. The slope is -2.149. **p**, Comparison of the directions of cell migration (red), cAMP increase gradient (blue), and cAMP decrease gradient (yellow).

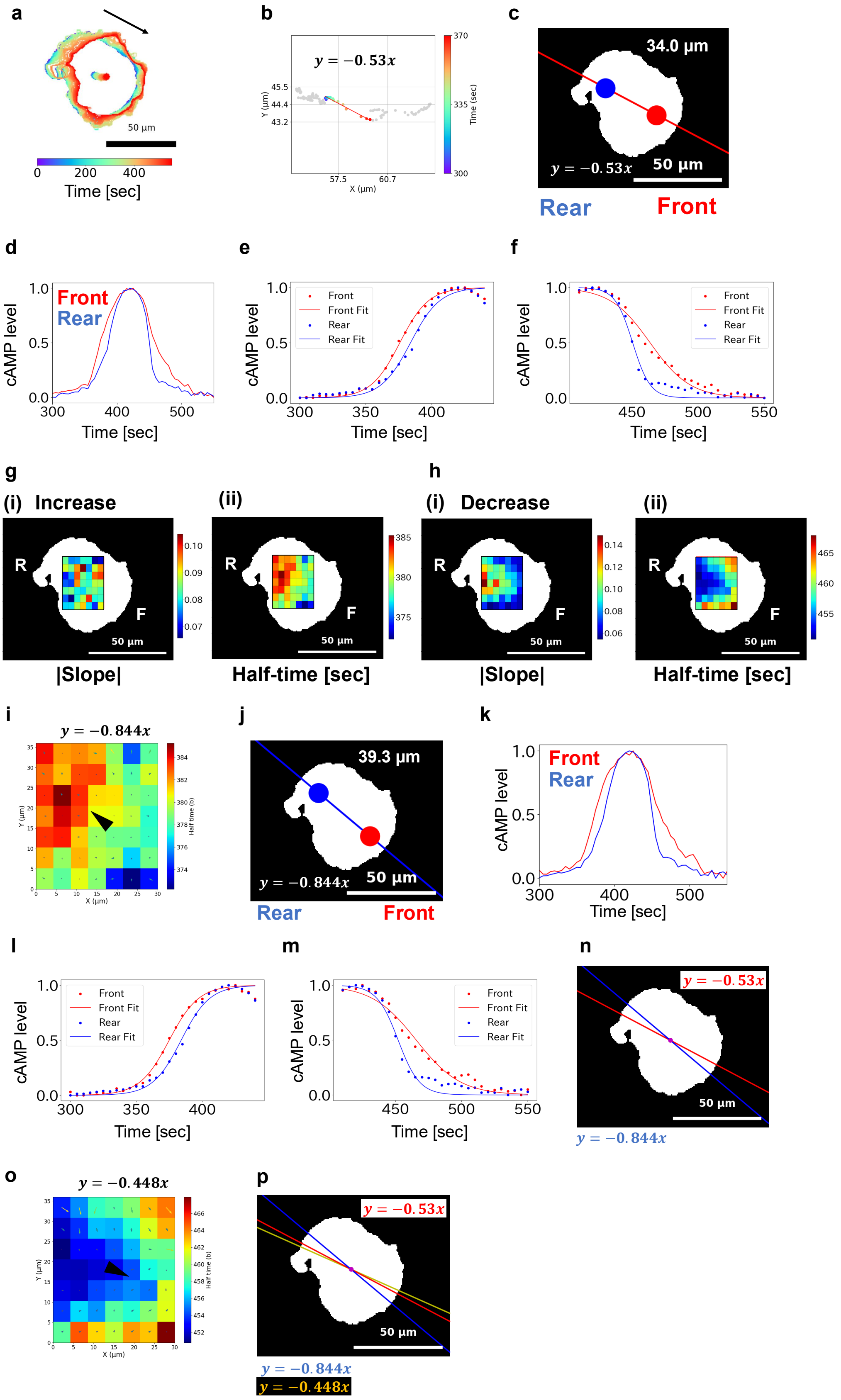

Supplementary Figure 8: Spatiotemporal analysis of cAMP with Flamindo2-RFP in another cell.

AX2 cells expressing Flamindo2-RFP were cultured with 10 µM blebbistatin under shaking

conditions for 8 days and then collected at $1×10^6$ cells/mL. After the cells were washed and starved with DB, confocal imaging was then initiated (40× objective, 5 sec intervals). **a**, Cell outline overlaid with the centroid trajectory. An arrow indicates the direction of migration. **b**, Linear approximation of the centroid trajectory between 300 and 370 sec. **c**, Definition of front (red) and rear (blue) regions for analysis based on the fitted trajectory. The regions were positioned ±15 μm from the centroid along the fitted line with a slope of −0.53 (diameter: 11 μm). **d**, Temporal changes in normalized intensity ratio in the front (red) and rear (blue) regions (x-axis: time [s]; y-axis: normalized intensity ratio). **e**, Sigmoid fitting of cAMP increase (red, front; blue, rear). **f**, Sigmoid fitting of cAMP decrease. **g**, (i) Heatmap of the slope parameter during cAMP increase; (ii) heatmap of the half-time parameter during cAMP increase. **h**, (i) Heatmap of the slope parameter during cAMP decrease; (ii) heatmap of the half-time parameter during cAMP decrease. **i**, Heatmap of the half-time parameter of cAMP increase. Δx = 4.286 μm; Δy = 5.143 μm. **j**, Based on the gradient direction of cAMP increase, front (red) and rear (blue) regions were defined along the fitted line at ±15 μm from the centroid with a slope of −0.844 (diameter: 11 μm). **k**, Temporal changes in normalized intensity ratio in the front (red) and rear (blue) regions. **l**, Sigmoid fitting of cAMP increase (red, front; blue, rear). **m**, Sigmoid fitting of cAMP decrease. **n**, Comparison of the direction of cell migration (red) with the gradient direction of cAMP increase (blue). **o**, Gradient direction of cAMP decrease determined in the same manner. The slope is -0.448. **p**, Comparison of the directions of cell migration (red), cAMP increase gradient (blue), and cAMP decrease gradient (yellow).

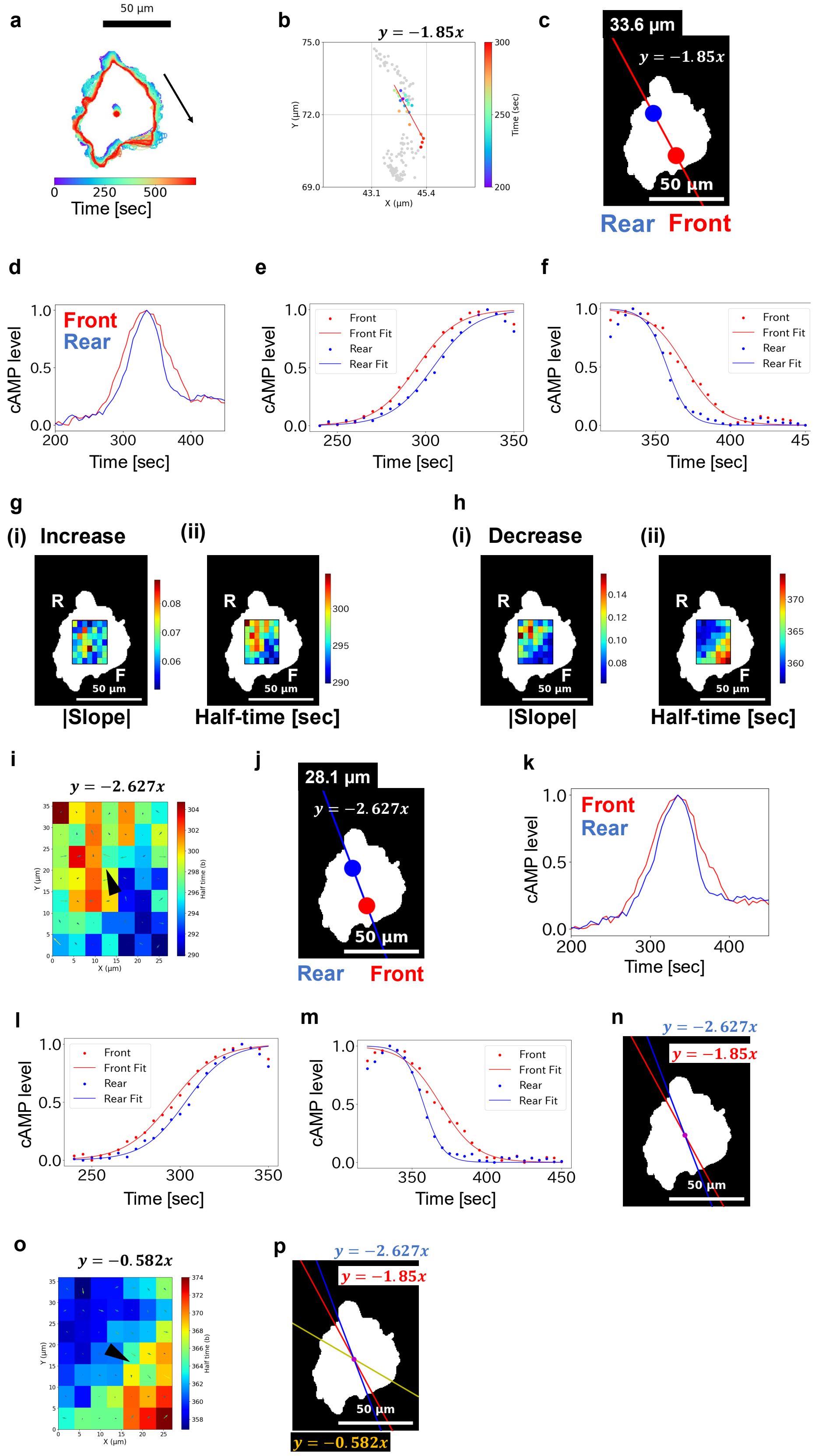

Supplementary Figure 9: Spatiotemporal analysis of cAMP with Flamindo2-RFP in a different cell.

AX2 cells expressing Flamindo2-RFP were cultured with 10 µM blebbistatin under shaking

conditions for 8 days and then collected at 1×10^6 cells/mL. After the cells were washed and starved with DB, confocal imaging was then initiated (40× objective, 5 sec intervals). **a**, Cell outline overlaid with the centroid trajectory. An arrow indicates the direction of migration. **b**, Linear approximation of the centroid trajectory between 200 and 300 sec. **c**, Definition of front (red) and rear (blue) regions for analysis based on the fitted trajectory. The regions were positioned ±8 μm from the centroid along the fitted line with a slope of -1.85 (diameter: 11 μm). **d**, Temporal changes in normalized intensity ratio in the front (red) and rear (blue) regions (x-axis: time [s]; y-axis: normalized intensity ratio). **e**, Sigmoid fitting of cAMP increase (red, front; blue, rear). **f**, Sigmoid fitting of cAMP decrease. **g**, (i) Heatmap of the slope parameter during cAMP increase; (ii) heatmap of the half-time parameter during cAMP increase. **h**, (i) Heatmap of the slope parameter during cAMP decrease; (ii) heatmap of the half-time parameter during cAMP decrease. **i**, Heatmap of the half-time parameter of cAMP increase. Δx = 3.857 μm; Δy = 5.143 μm. **j**, Based on the gradient direction of cAMP increase, front (red) and rear (blue) regions were defined along the fitted line at ±5 μm from the centroid with a slope of -2.627 (diameter: 11 μm). **k**, Temporal changes in normalized intensity ratio in the front (red) and rear (blue) regions. **l**, Sigmoid fitting of cAMP increase (red, front; blue, rear). **m**, Sigmoid fitting of cAMP decrease. **n**, Comparison of the direction of cell migration (red) with the gradient direction of cAMP increase (blue). **o**, Gradient direction of cAMP decrease determined in the same manner. The slope is -0.582. **p**, Comparison of the directions of cell migration (red), cAMP increase gradient (blue), and cAMP decrease gradient (yellow).

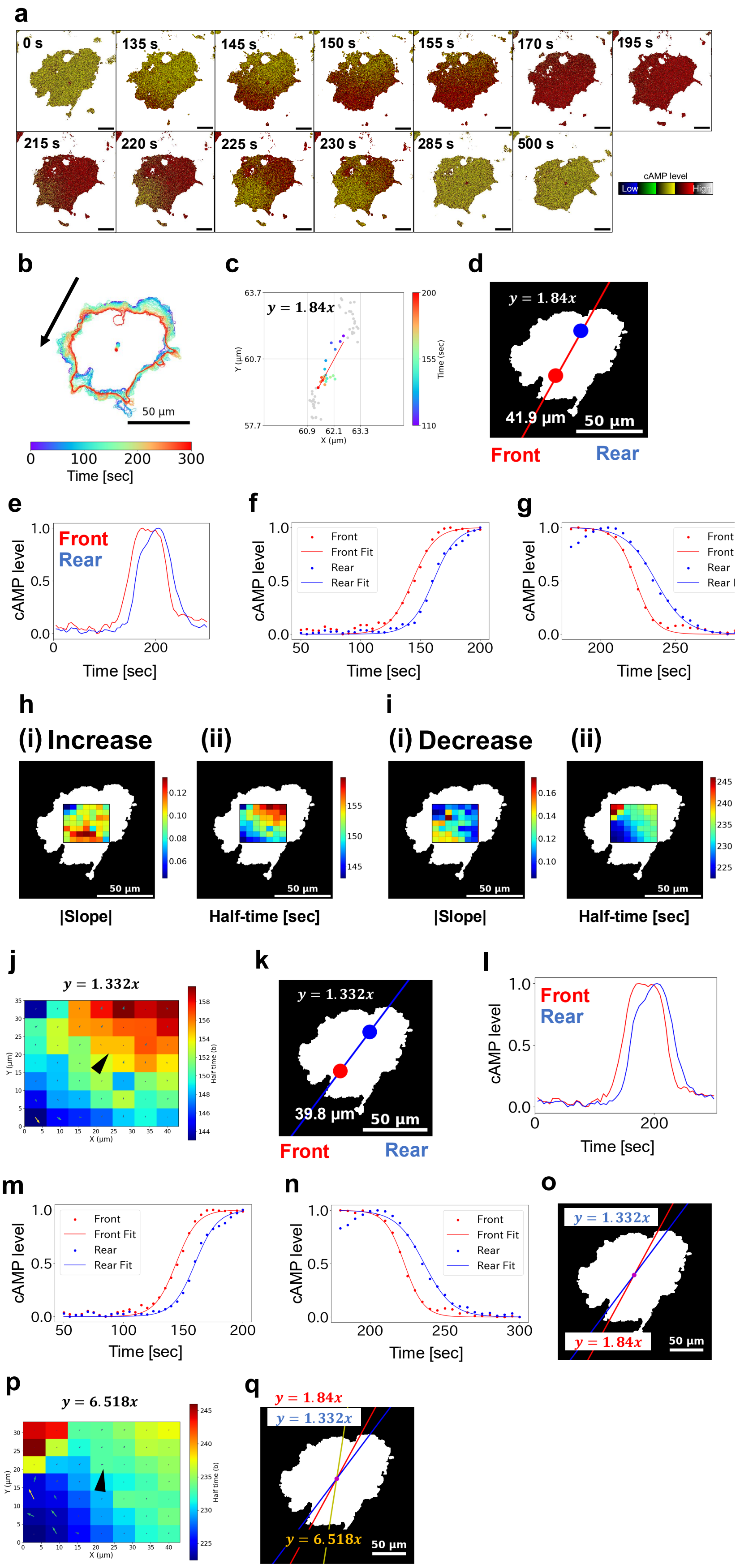

Supplementary Figure 10: Temporal analysis of front–rear polarity in cAMP signaling.

AX2 cells expressing Flamindo2-RFP were cultured with 10 µM blebbistatin under shaking

conditions for 6 days and then collected at 1×10^6 cells/mL. After the cells were washed and starved with DB, confocal imaging was then initiated (40× objective, 5 sec intervals). **a**, Time-lapse fluorescence ratio images (RFP/Flamindo2) of a giant cell. The number in the top left indicates the elapsed time from the start of imaging. Scale bars: 20 μm. **b**, Cell outline overlaid with the centroid trajectory. An arrow indicates the direction of migration. **c**, Linear approximation of the centroid trajectory between 110 and 200 sec. **d**, Definition of front (red) and rear (blue) regions for analysis based on the fitted trajectory. The regions were positioned ±10 μm from the centroid along the fitted line with a slope of 1.84 (diameter: 11 μm). **e**, Temporal changes in normalized intensity ratio in the front (red) and rear (blue) regions (x-axis: time [s]; y-axis: normalized intensity ratio). **f**, Sigmoid fitting of cAMP increase (red, front; blue, rear). **g**, Sigmoid fitting of cAMP decrease. **h**, (i) Heatmap of the slope parameter during cAMP increase; (ii) heatmap of the half-time parameter during cAMP increase. **i**, (i) Heatmap of the slope parameter during cAMP decrease; (ii) heatmap of the half-time parameter during cAMP decrease. **j**, Heatmap of the half-time parameter of cAMP increase. Δx = 6.143 μm; Δy = 5.000 μm. **k**, Based on the gradient direction of cAMP increase, front (red) and rear (blue) regions were defined along the fitted line at ±12 μm from the centroid with a slope of 1.332 (diameter: 11 μm). **l**, Temporal changes in normalized intensity ratio in the front (red) and rear (blue) regions. **m**, Sigmoid fitting of cAMP increase (red, front; blue, rear). **n**, Sigmoid fitting of cAMP decrease. **o**, Comparison of the direction of cell migration (red) with the gradient direction of cAMP increase (blue). **p**, Gradient direction of cAMP decrease determined in the same manner. The slope is 6.518. **q**, Comparison of the directions of cell migration (red), cAMP increase gradient (blue), and cAMP decrease gradient (yellow).

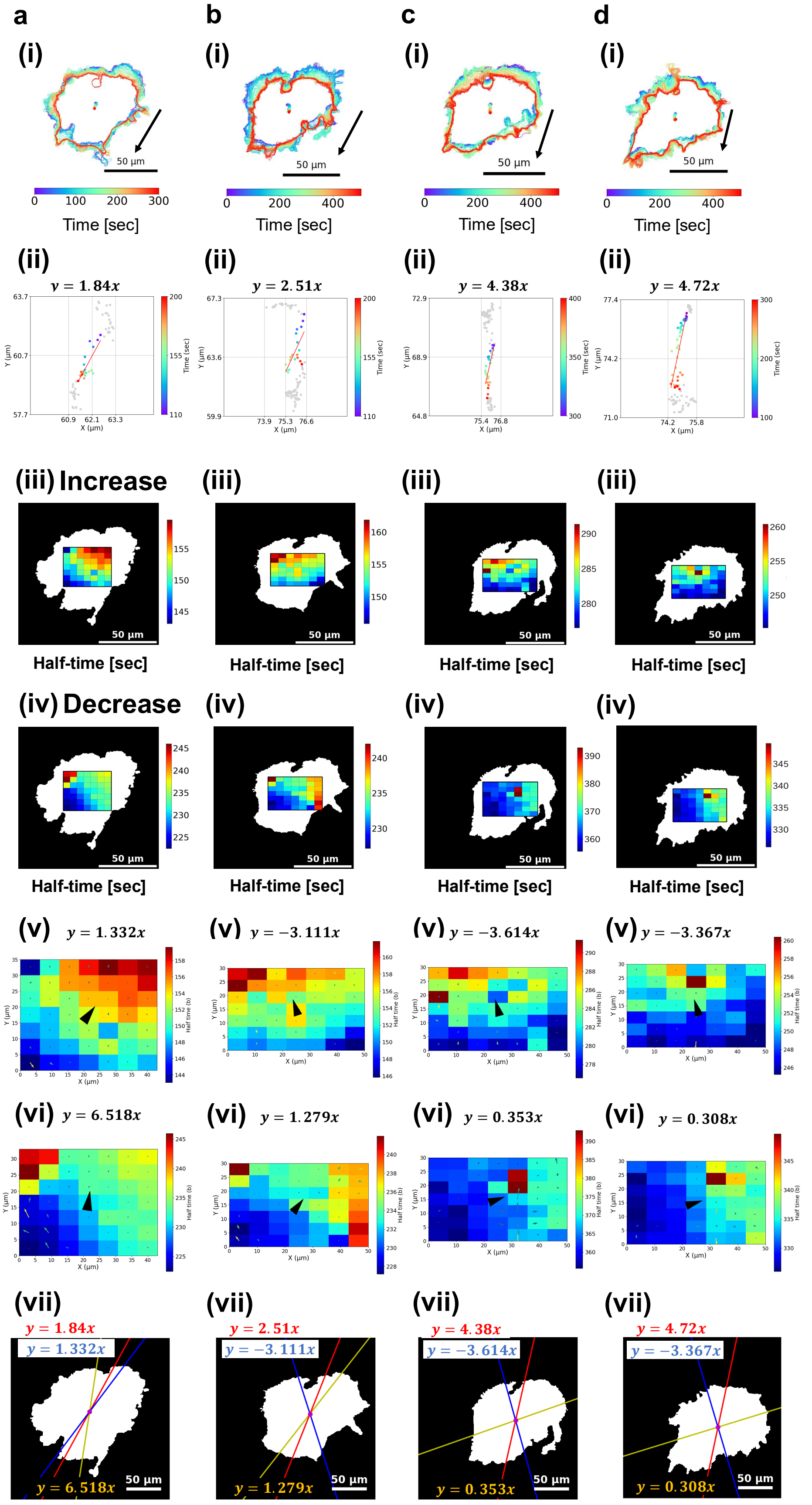

Supplementary Figure 11: Temporal analysis of front–rear polarity with heatmaps.

For the cell shown in Fig. S10, the directionality of cell migration and cAMP signaling was

analyzed under conditions in which intracellular cAMP levels continuously changed. **a–d**, Panels a–d show the analysis of the 1st, 2nd, 3rd, and 4th cAMP signaling events, respectively. (i), Cell outlines overlaid with centroid trajectories. Arrows indicate the direction of migration. (ii), Linear approximation of the centroid trajectories. (iii) Heatmaps of the half-time parameter during cAMP increase. (iv) Heatmaps of the half-time parameter during cAMP decrease. Gradient direction of cAMP increase (v) and decrease (vi). (vii) Comparison of the directions of cell migration (red), cAMP increase gradient (blue), and cAMP decrease gradient (yellow).

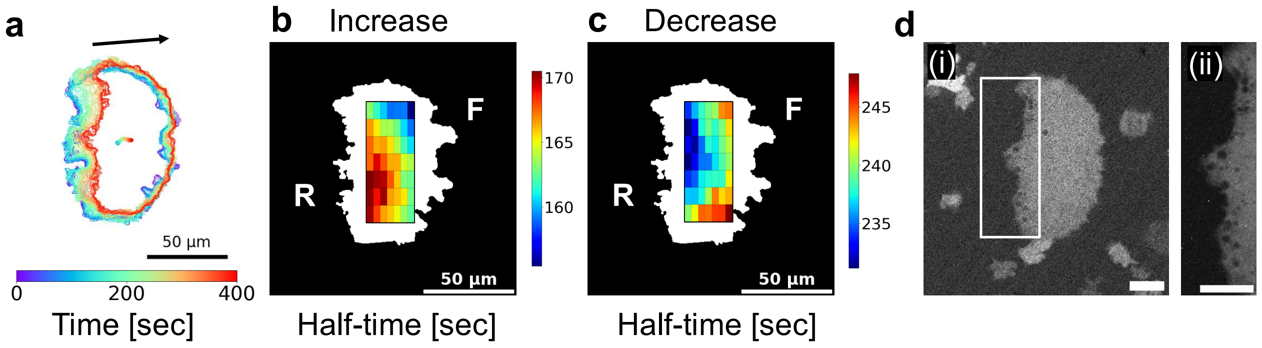

Supplementary Figure 12: Observation of vesicle localization at the rear of giant cells. **a**, Trajectory of a Flamindo2-RFP giant cell (cultured for 6 days). Scale bar: 50 µm. **b**, Heatmap of half-time during the cAMP increase phase. **c**, Heatmap of half-time during the cAMP decrease phase. **d**, Localization of vesicles at the cell rear. The white box indicates magnified area shown in (ii). Scale bars: 20 µm.

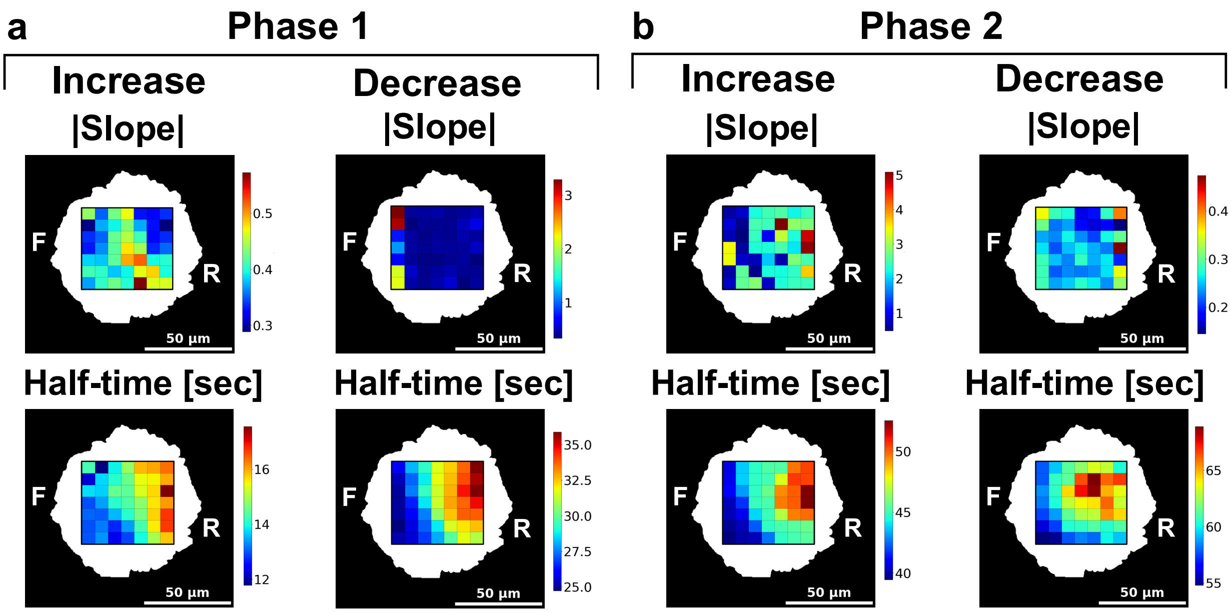

Supplementary Figure 13: Analysis of Ca^{2+} signaling dynamics in a giant cell.
Analysis of the giant cell in Fig. 7. **a**, First-phase Ca^{2+} signal analysis: spatial grid (7×7 within a 54 µm × 50 µm rectangle) of the increase phase, heatmap of the slope parameter, and heatmap of half-time parameter derived from sigmoid fitting (left panels). Heatmaps of the decrease phase during the first Ca^{2+} wave: slope and half-time (right panels). **b**, Second-phase Ca^{2+} signal analysis: increase phase heatmaps, slope and half-time (left panels); decrease phase heatmaps, slope and half-time (right panels).

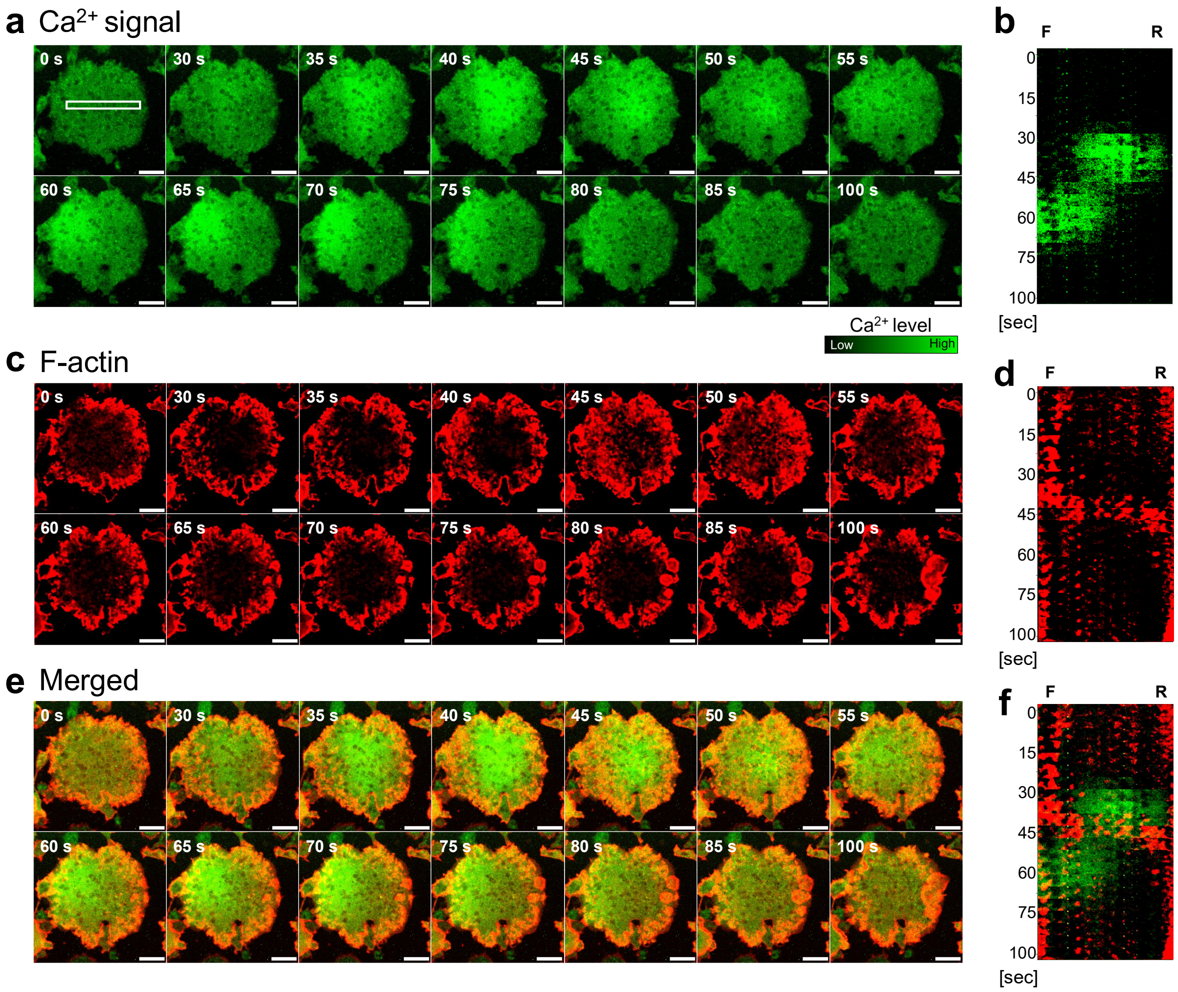

Supplementary Figure 14: Coordination between Ca^{2+} signals and F-actin dynamics in another cell.

AX2 cells co-expressing GCaMP6s and Lifeact14-mScarletI were cultured for 6 days under shaking conditions with 10 μM blebbistatin. After the cells were starved with DB, imaging was performed using confocal microscopy (40× objective; 5 sec intervals). **a**, Fluorescence time-lapse images of GCaMP6s in a giant cell. Time after imaging onset is shown in the top-right corner. Scale bars: 20 μm. **b**, Kymograph of Ca^{2+} signal intensity within the white rectangle indicated in (a). Vertical axis: time [sec]. **c**, Time-lapse images of actin waves (Lifeact14-mScarletI channel) in the same cell. Scale bars: 20 μm. **d**, Kymograph of actin wave dynamics along the same region. Vertical axis: time [sec]. **e**, Merged fluorescence images showing Ca^{2+} signals (GCaMP6s, green) and actin waves (Lifeact14-mScarletI, red). Scale bars: 20 μm. **f**, Merged kymograph of Ca^{2+} signals and actin waves.

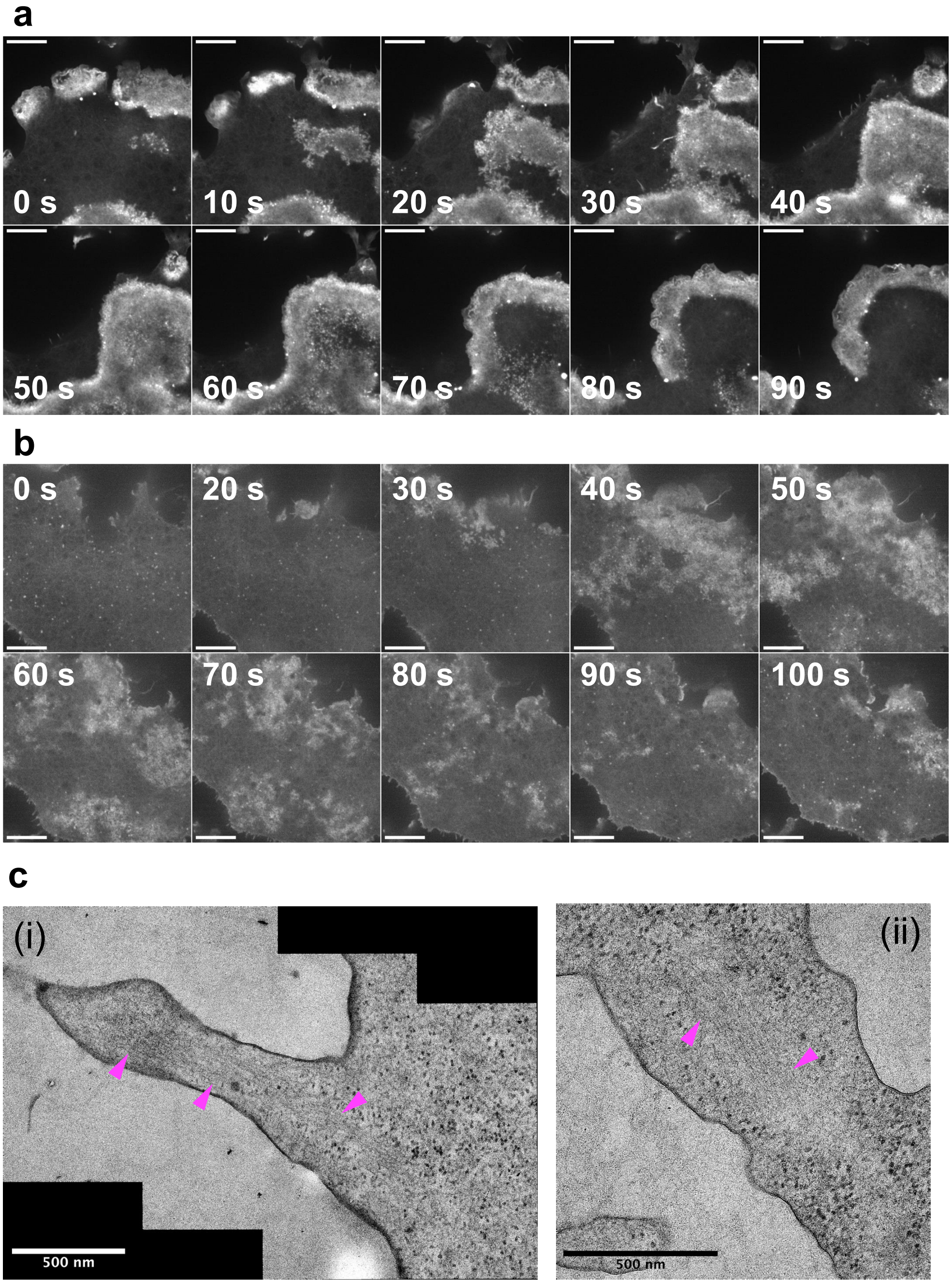

Supplementary Figure 15: Visualization of actin dynamics in giant cells using super-resolution microscopy and electron microscopy.

AX2 cells expressing Lifeact14-mScarletI were cultured with 10 μM blebbistatin under shaking conditions for 6 days. Imaging using confocal microscopy (40× objective, 5 sec intervals) was initiated the cells were starved for 7.5 hours with DB. **a**, Representative time-lapse Lifeact14-mScarletI fluorescence images of a giant cell. Numbers indicate time elapsed from the beginning of imaging. Scale bars: 10 μm. **b**, Representative time-lapse Lifeact14-mScarletI fluorescence images of another giant cell. Scale bars: 10 μm. **c**, Actin bundle structures (magenta arrowheads) were visualized by combining the giant cell preparation method with electron microscopy. Scale bars: 500 nm.

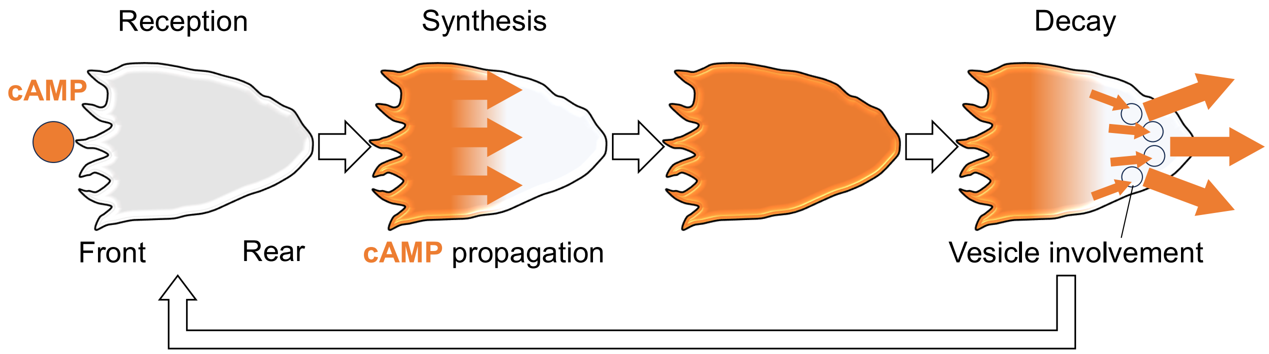

Supplementary Figure 16: Schematic overview of the cAMP signaling mechanism. The incoming cAMP signal defines the cell front. cAMP synthesis initiated at the front propagates toward the rear, where rear-localized vesicles promote extracellular release, causing intracellular cAMP levels to decrease from the back. Repetition of this cycle generates the characteristic rise-and-fall dynamics of intracellular cAMP levels during signal relay.

Supplementary Video Legends

Supplementary Video 1.
Time-lapse fluorescence imaging of cAMP signal relay was performed in AX2 cells expressing Flamindo2. Giant cells were constructed by blebbistatin treatment and shaking culture. Scale bar: 100 μm. Corresponds to Supplementary Fig. 2a.

Supplementary Video 2.
Time-lapse fluorescence imaging of Ca^{2+} oscillations in *Dictyostelium* cells expressing GCaMP6s. Giant cells were constructed by blebbistatin treatment and shaking culture. Scale bar: 50 μm. Corresponds to Supplementary Fig. 2b.

Supplementary Video 3.
Time-lapse fluorescence imaging of front-to-rear propagation of intracellular cAMP signaling in a migrating giant cell expressing Flamindo2. Scale bar: 20 μm. Corresponds to Fig. 2.

Supplementary Video 4.
Spatiotemporal variations in cAMP signal initiation and decay in *Dictyostelium* cells expressing Flamindo2-RFP. Scale bar: 20 μm. Corresponds to Supplementary Fig. 10a and 11a.

Supplementary Video 5.
Time-lapse fluorescence imaging of cAMP signaling in *Dictyostelium* cells expressing Flamindo2-RFP. Scale bar: 20 μm. Corresponds to Supplementary Fig. 11b.

Supplementary Video 6.
Time-lapse fluorescence imaging of cAMP signaling in *Dictyostelium* cells expressing Flamindo2-RFP. Scale bar: 20 μm. Corresponds to Supplementary Fig. 11c.

Supplementary Video 7.
Time-lapse fluorescence imaging of cAMP signaling in *Dictyostelium* cells expressing Flamindo2-RFP. Scale bar: 20 μm. Corresponds to Supplementary Fig. 11d.

Supplementary Video 8.
Micropipette-based cAMP stimulation reveals that signal synthesis initiates near the stimulus. The asterisk indicates the position of the needle tip used to apply cAMP. Scale bar:

50 µm. Corresponds to Fig. 4a.

Supplementary Video 9.
Micropipette-based cAMP stimulation reveals that signal synthesis initiates near the stimulus. Scale bar: 50 µm. Corresponds to Fig. 4b, c.

Supplementary Video 10.
Micropipette-based cAMP stimulation reveals that signal synthesis initiates near the stimulus. The asterisk indicates the position of the needle tip used to apply cAMP. Scale bar: 50 µm. Corresponds to Fig. 4d.

Supplementary Video 11.
Micropipette-based cAMP stimulation reveals that signal synthesis initiates near the stimulus. Scale bar: 50 µm. Corresponds to Fig. 4e.

Supplementary Video 12.
Biphasic Ca^{2+} dynamics in response to cAMP in a giant cell. GCaMP6s imaging captures dual Ca^{2+} peaks in each cAMP cycle. Scale bar: 20 µm. Corresponds to Fig. 6a.

Supplementary Video 13.
Spatiotemporal coupling of Ca^{2+} signals and actin wave propagation. Simultaneous imaging of GCaMP6s and Lifeact14-mScarletI shows that actin waves emerge following the reduction of Ca^{2+} levels, indicating inverse coordination between signaling and cytoskeletal dynamics. Scale bar: 20 µm. Corresponds to Fig. 7.

Supplementary Video 14.
Super-resolution imaging of actin structures in a giant *Dictyostelium* cell. SoRA spinning-disk confocal microscopy reveals fine actin meshworks in enlarged migrating cells. Scale bar: 20 µm. Corresponds to Supplementary Fig. 15a.

Supplementary Video 15.
Super-resolution imaging of actin structures in a giant *Dictyostelium* cell. Scale bar: 20 µm. Corresponds to Supplementary Fig. 15b.

Table S1

centroid trajectory

Data No	Distance [µm]	Increase				Decrease			
		Front		Rear		Front		Rear	
		Slope	Half time [sec]	Slope	Half time [sec]	Slope	Half time [sec]	Slope	Half time [sec]
No1	43.4	0.12	94.6	0.166	107.9	-0.166	228.7	-0.199	226.7
No2	36.1	0.0673	403.3	0.0616	415.4	-0.249	491.4	-0.267	490.6
No3	56.9	0.0994	296.2	0.0829	316.9	-0.112	383.7	-0.164	377.2

cAMP gradient

Data No	Distance [µm]	Increase				Decrease			
		Front		Rear		Front		Rear	
		Slope	Half time [sec]	Slope	Half time [sec]	Slope	Half time [sec]	Slope	Half time [sec]
No1	42.7	0.123	94.9	0.162	107.8	-0.169	228.6	-0.199	226.7
No2	35.6	0.0697	403.4	0.0604	415.5	-0.239	491.8	-0.271	490.5
No3	50.9	0.115	297.2	0.0685	316.1	-0.122	383.5	-0.203	375.7

Table S2

Inccrease (centroid trajectory)

Data No	Distance [μm]	Half time difference between Front and Rear [sec]	Propagation velocity [μm/sec]	Diffusion coefficient [$μm^2$/sec]
No1	43.4	13.3	3.26	70.8
No2	36.1	12.1	2.98	53.9
No3	56.9	20.7	2.75	78.2
Average	-	-	3	67.6

Inccrease (cAMP gradient)

Data No	Distance [μm]	Half time difference between Front and Rear [sec]	Propagation velocity [μm/sec]	Diffusion coefficient [$μm^2$/sec]
No1	42.7	12.9	3.31	70.7
No2	35.6	12.1	2.94	52.4
No3	50.9	18.9	2.69	68.5
Average	-	-	2.98	63.9

Table S3

Deccrease (centroid trajectory)

Data No	Distance [μm]	Half time difference between Front and Rear [sec]
No1	43.4	2
No2	36.1	0.8
No3	56.9	6.5

Deccrease (cAMP gradient)

Data No	Distance [μm]	Half time difference between Front and Rear [sec]
No1	42.7	1.9
No2	35.6	1.3
No3	50.9	7.8

Table S4

centroid trajectory

Data No	Distance [μm]	Increase				Decrease			
		Front		Rear		Front		Rear	
		Slope	Half time [sec]	Slope	Half time [sec]	Slope	Half time [sec]	Slope	Half time [sec]
No1	40.2	0.0608	100.5	0.0621	121.3	-0.0719	206.6	-0.119	185.4
No2	34	0.0917	375.9	0.0899	384	-0.0663	463.8	-0.151	450.6
No3	33.6	0.0858	294.8	0.0833	304.2	-0.0841	370.1	-0.141	357.9

cAMP gradient

Data No	Distance [μm]	Increase				Decrease			
		Front		Rear		Front		Rear	
		Slope	Half time [sec]	Slope	Half time [sec]	Slope	Half time [sec]	Slope	Half time [sec]
No1	40.2	0.0617	101.1	0.0651	120.9	-0.0749	205.5	-0.12	186.2
No2	39.3	0.0894	375	0.0922	384	-0.06	465.8	-0.124	452.1
No3	28.1	0.0768	295.2	0.0836	303.1	-0.0825	369	-0.156	357.8

Table S5

Inccrease (centroid trajectory)

Data No	Distance [μm]	Half time difference between Front and Rear [sec]	Propagation velocity [μm/sec]	Diffusion coefficient [$μm^2$/sec]
No1	40.2	20.8	1.93	38.8
No2	34	8.1	4.2	71.4
No3	33.6	9.4	3.57	60.1
Average	-	-	3.23	56.8

Inccrease (cAMP gradient)

Data No	Distance [μm]	Half time difference between Front and Rear [sec]	Propagation velocity [μm/sec]	Diffusion coefficient [$μm^2$/sec]
No1	40.2	19.8	2.03	40.8
No2	39.3	9	4.37	85.8
No3	28.1	7.9	3.56	50
Average	-	-	3.32	58.9

Table S6

Deccrease (centroid trajectory)

Data No	Distance [μm]	Half time difference between Front and Rear [sec]
No1	40.2	21.2
No2	34	13.2
No3	33.6	12.2

Deccrease (cAMP gradient)

Data No	Distance [μm]	Half time difference between Front and Rear [sec]
No1	40.2	19.3
No2	39.3	13.7
No3	28.1	11.2

Table S7 **Strains used in this study**

Strain	Characteristics	Background	Source or reference
AX2	Wild type	AX2	Lab stock
Flamindo2, H2B-RFP / AX2	(neoR, bsR)	AX2	NBRP-nenkin (ID: S90795)
Flamindo2-mRFPmars / AX2	(neoR)	AX2	Hayashida *et al*., 2025
GCaMP6s / AX2	(hygR)	AX2	This study
GCaMP6s, Lifeact14-mScarletI / AX2	(neoR, hygR)	AX2	This study
PH-RFP / AX2	(neoR)	AX2	This study

Table S8 **Plasmids used in this study**

Plasmid	Characteristics	Backbone	Source or reference
pHK12neo / Flamindo2	Flamindo2	pHK12neo (neoR)	Hashimura *et al* ., 2019<br>NBRP-nenkin (ID: S90789)
pHK12neo / Flamindo2-RFP	Flamindo2-mRFPmars	pHK12neo (neoR)	Hayashida *et al* ., 2025
pHK12neo/ GCaMP6s	Dd-GCaMP6s	pHK12neo (neoR)	Hashimura *et al* ., 2022<br>NBRP-nenkin (ID: G90729)
pDM358/ GCaMP6s	Dd-GCaMP6s	pDM358 (hygR)	This study
pDM358/ Lifeact14-mScarletI	Lifeact14-mScarletI	pDM358 (hygR)	This study
pHK12neo/ PH-RFP	PH(Akt)-mRFPmars	pHK12neo (neoR)	NBRP-nenkin (ID: G90174)

Table S9 **Primers used in this study**

Primer	Sequence
BglII-Lifeact14-mSc_Fw	AAAGATCTAAAAAATGGGTGTTGCTGATCTTATTAAGAAATTTGAAAGTATTAGTAGTAAAGGAGAAGCTGT
mSc-SpeI_Rv	AAACTAGTTATTTGTATAGTTCATCCATGC
