# Supplementary material for: Establishing functional giant Dictyostelium cells reveals front–rear polarity in intracellular signaling
Source: Commun Biol. 2026 Jan 21;9:71. doi: 10.1038/s42003-025-09505-7 (PMC12824319; doi:10.1038/s42003-025-09505-7)
Supplement: Supplementary file 3 — Description of Additional Supplementary Files [file 42003_2025_9505_MOESM3_ESM.pdf]

Description of Additional Supplementary File

File name: Supplementary Video 1

Description: Time-lapse fluorescence imaging of cAMP signal relay was performed in AX2 cells expressing Flamindo2. Giant cells were constructed by blebbistatin treatment and shaking culture. Scale bar: 100 μm. Corresponds to Supplementary Fig. 2a.

File name: Supplementary Video 2

Description: Time-lapse fluorescence imaging of Ca2+ oscillations in Dictyostelium cells expressing GCaMP6s. Giant cells were constructed by blebbistatin treatment and shaking culture. Scale bar: 50 μm. Corresponds to Supplementary Fig. 2b.

File name: Supplementary Video 3

Description: Time-lapse fluorescence imaging of front-to-rear propagation of intracellular cAMP signaling in a migrating giant cell expressing Flamindo2. Scale bar: 20 μm. Corresponds to Fig. 2.

File name: Supplementary Video 4

Description: Spatiotemporal variations in cAMP signal initiation and decay in Dictyostelium cells expressing Flamindo2-RFP. Scale bar: 20 μm. Corresponds to Supplementary Fig. 10a and 11a.

File name: Supplementary Video 5

Description: Time-lapse fluorescence imaging of cAMP signaling in Dictyostelium cells expressing Flamindo2-RFP. Scale bar: 20 μm. Corresponds to Supplementary Fig. 11b.

File name: Supplementary Video 6

Description: Time-lapse fluorescence imaging of cAMP signaling in Dictyostelium cells expressing Flamindo2-RFP. Scale bar: 20 μm. Corresponds to Supplementary Fig. 11c.

File name: Supplementary Video 7

Description: Time-lapse fluorescence imaging of cAMP signaling in Dictyostelium cells expressing Flamindo2-RFP. Scale bar: 20 μm. Corresponds to Supplementary Fig. 11d.

File name: Supplementary Video 8

Description: Micropipette-based cAMP stimulation reveals that signal synthesis initiates near the stimulus. The asterisk indicates the position of the needle tip used to apply cAMP. Scale bar: 50 μm. Corresponds to Fig. 4a.

File name: Supplementary Video 9

Description: Micropipette-based cAMP stimulation reveals that signal synthesis initiates near the stimulus. Scale bar: 50 μm. Corresponds to Fig. 4b, c.

File name: Supplementary Video 10

Description: Micropipette-based cAMP stimulation reveals that signal synthesis initiates near the stimulus. The asterisk indicates the position of the needle tip used to apply cAMP. Scale bar: 50 μm. Corresponds to Fig. 4d.

File name: Supplementary Video11

Description: Micropipette-based cAMP stimulation reveals that signal synthesis initiates near the stimulus. Scale bar: 50 μm. Corresponds to Fig. 4e.

File name: Supplementary Video 12

Description: Biphasic Ca2+ dynamics in response to cAMP in a giant cell. GCaMP6s imaging captures dual Ca2+ peaks in each cAMP cycle. Scale bar: 20 μm. Corresponds to Fig. 6a.

File name: Supplementary Video 13

Description: Spatiotemporal coupling of Ca2+ signals and actin wave propagation. Simultaneous imaging of GCaMP6s and Lifeact14-mScarletI shows that actin waves emerge following the reduction of Ca2+ levels, indicating inverse coordination between signaling and cytoskeletal dynamics. Scale bar: 20 μm. Corresponds to Fig. 7.

File name: Supplementary Video 14

Description: Super-resolution imaging of actin structures in a giant Dictyostelium cell. SoRA spinningdisk confocal microscopy reveals fine actin meshworks in enlarged migrating cells. Scale bar: 20 μm. Corresponds to Supplementary Fig. 15a.

File name: Supplementary Video 15

Description: Super-resolution imaging of actin structures in a giant Dictyostelium cell. Scale bar: 20 μm. Corresponds to Supplementary Fig. 15b.
